# Lactose Intolerance in Adults: Biological Mechanism and Dietary Management

**DOI:** 10.3390/nu7095380

**Published:** 2015-09-18

**Authors:** Yanyong Deng, Benjamin Misselwitz, Ning Dai, Mark Fox

**Affiliations:** 1 Department of Gastroenterology, Sir Run Run Shaw Hospital, School of Medicine, Zhejiang University, 3 East Qingchun Road, 310016 Hangzhou, China; E-Mails: dengyanyong@163.com (Y.D.); ndaicn@yahoo.com (N.D.); 2 Neurogastroenterology and Motility Research Group, Department of Gastroenterology and Hepatology, Division of Gastroenterology & Hepatology, University Hospital Zürich, Zürich CH-8091, Switzerland; E-Mail: benjamin.misselwitz@usz.ch; 3 Division of Gastroenterology, St. Claraspital, 4058 Basel, Switzerland

**Keywords:** lactose intolerance, lactase deficiency, lactose malabsorption, FODMAP, genetic test, hydrogen breath test, irritable bowel syndrome

## Abstract

Lactose intolerance related to primary or secondary lactase deficiency is characterized by abdominal pain and distension, borborygmi, flatus, and diarrhea induced by lactose in dairy products. The biological mechanism and lactose malabsorption is established and several investigations are available, including genetic, endoscopic and physiological tests. Lactose intolerance depends not only on the expression of lactase but also on the dose of lactose, intestinal flora, gastrointestinal motility, small intestinal bacterial overgrowth and sensitivity of the gastrointestinal tract to the generation of gas and other fermentation products of lactose digestion. Treatment of lactose intolerance can include lactose-reduced diet and enzyme replacement. This is effective if symptoms are only related to dairy products; however, lactose intolerance can be part of a wider intolerance to variably absorbed, fermentable oligo-, di-, monosaccharides and polyols (FODMAPs). This is present in at least half of patients with irritable bowel syndrome (IBS) and this group requires not only restriction of lactose intake but also a low FODMAP diet to improve gastrointestinal complaints. The long-term effects of a dairy-free, low FODMAPs diet on nutritional health and the fecal microbiome are not well defined. This review summarizes recent advances in our understanding of the genetic basis, biological mechanism, diagnosis and dietary management of lactose intolerance.

## 1. Lactose and Lactase

Lactose is a disaccharide consisting of galactose bound to glucose and is of key importance in animal life as the main source of calories from milk of all mammals, all except the sea lion. Intestinal absorption of lactose requires hydrolysis to its component monosaccharides by the brush-border enzyme lactase. From week 8 of gestation, lactase activity can be detected at the mucosal surface in the human intestine. Activity increases until week 34 and lactase expression is at its peak by birth. The ability to digest lactose during the period of breast-feeding is essential to the health of the infant as demonstrated by congenital lactase deficiency that is fatal if not recognized very early after birth. However, following the first few months of life, lactase activity starts to decrease (lactase non-persistence). In most humans, this activity declines following weaning to undetectable levels as a consequence of the normal maturational down-regulation of lactase expression [1]. The exceptions to this rule are the descendants of populations that traditionally practice cattle domestication maintain the ability to digest milk and other dairy products into adulthood. The frequency of this “lactase persistence trait” is high in northern European populations (>90% in Scandinavia and Holland), decreases in frequency across southern Europe and the Middle East (~50% in Spain, Italy and pastoralist Arab populations) and is low in Asia and most of Africa (~1% in Chinese, ~5%–20% in West African agriculturalists); although it is common in pastoralist populations from Africa (~90% in Tutsi, ~50% in Fulani) [2].

## 2. Genetics of Lactase Persistence

**Figure 1:**
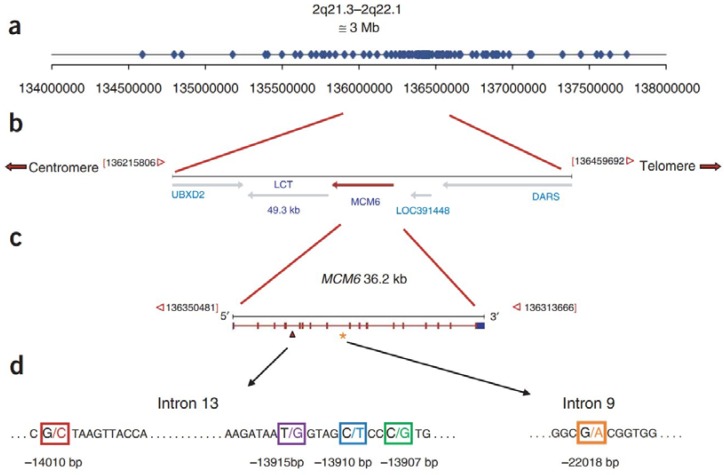
Map of the lactase (LCT) and minichromosome maintenance 6 (MCM6) gene region and location of genotyped single nucleotide polymorphisms (SNPs). (**a**) Distribution of 123 SNPs included in genotype analysis; (**b**) map of the LCT and MCM6 gene region; (**c**) map of the MCM6 gene; and (**d**) location of lactase persistence-associated SNPs within introns 9 and 13 of the MCM6 gene in African and European populations [12].

Lactase persistence is thought to be related to the domestication of dairy cattle during the last 10,000 years. Lactase persistence is inherited as a dominant Mendelian trait [3]. Adult expression of the gene encoding lactase (LCT), located on 2q21 appears to be regulated by *cis*-acting elements [4]. A linkage disequilibrium (LD) and haplotype analysis of Finnish pedigrees identifies two single single nucleotide polymorphisms (SNPs) associated with the lactase persistence trait: C/T-13910 and G/A-22018, located ~14 kb and ~22 kb upstream of LCT, respectively, within introns 9 and 13 of the adjacent minichromosome maintenance 6 (MCM6) gene [3]. The T-13910 and A-22018 alleles are 100% and 97% associated with lactase persistence, respectively, in the Finnish study, and the T-13910 allele is ~86%–98% associated with lactase persistence in other European populations [5,6,7]. The genotype in China is C/C-13910, and no SNP associated with lactase persistence has been identified in the lactase gene regulatory sequence [8,9]. However, there are several lactase gene single nucleotide polymorphisms of this kind in other populations. Lactase persistence is mediated by G-13915 in Saudi Arabia [10], in African tribes by the G-14010, G-13915, and G-13907 polymorphism (Figure 1) [11,12]. Thus, lactase persistence developed several times independently in human evolution in different areas of the world [11]. Multiple independent variants have allowed various human populations to quickly modify LCT expression and have been strongly conserved in adult milk-consuming populations, emphasizing the importance of regulatory mutations in recent human evolution [13]. In adult patients with homozygous lactase persistence, enzyme levels at the jejunal brush border are 10-times higher than for patients with homozygous non-persistence, and heterozygous individuals [14].

## 3. Biological Mechanism of Lactose Intolerance

About two thirds of the World’s population undergoes a genetically programmed decrease in lactase synthesis after weaning (primary lactase deficiency) [15,16]. Additionally, in individuals with lactase persistence the occurrence of gastrointestinal infection, inflammatory bowel disease, abdominal surgery and other health issues can also cause a decrease in lactase activity (secondary lactase deficiency). Both conditions must be distinguished from congenital lactase deficiency, which is an extremely rare disease of infancy with approximately 40 cases having been reported, mainly in Finland [2].

**Figure 2:**
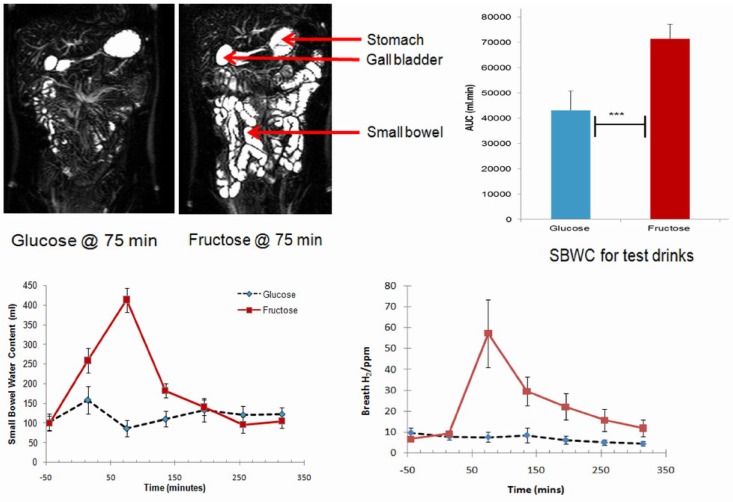
Small bowel water content (SBWC) and breath hydrogen (H2) concentrations after drinking each of the drinks: glucose and fructose. The time of drinking (*t* = 0 min) is highlighted in the chart. Values of SBWC are mean volume (mL) ± s.e.m (standard error of mean). Values of H2 are mean concentration (p.p.m.) ± s.e.m. Figure modified from Murray *et al*. [21].

Whatever the cause, lactase deficiency results in unabsorbed lactose being present in the intestinal tract, which has effects that can lead to symptoms of lactose intolerance in susceptible individuals [17]. First, the increased osmotic load increases the intestinal water content. Second, lactose is readily fermented by the colonic microbiome leading to production of short chain fatty acids and gas (mainly hydrogen (H_2_), carbon dioxide (CO_2_), and methane (CH_4_)). These biological processes are present also for other poorly-absorbed, fermentable oligosaccharides, disaccharides, monosaccharides, and polyols (FODMAPs) that are ubiquitous in the diet [18,19]. Double-blind, cross-over studies in healthy volunteers applied scintigraphy or magnetic resonance imaging to document oro-cecal transit time together with breath testing to assess fermentation of the substrate. Fructose (a disaccharide similar to lactose) was seen to increase small bowel water, accelerate oro-caecal transit time (OCTT) and trigger a sharp increase in breath hydrogen production (Figure 2), whereas 30 g glucose (a well-absorbed control) had no effect [20,21]. It should be noted that these effects are seen for poorly-absorbed, fermentable disaccharides both in health and in patients with gastrointestinal disease [22,23,24]. Long-chain carbohydrates (e.g., fructans, cellulose (“dietary fiber”)) that are not digested or absorbed by the small intestine have less impact on small bowel transit than short-chain carbohydrates; however, fermentation of this material in the large bowel produces similar effects on colonic function [21]. 

Malabsorption is a necessary precondition for lactose or FODMAP intolerance; however, the two are not synonymous and the causes of symptoms must be considered separately [25]. The threshold for dietary lactose tolerance is dependent on several factors including the dose consumed, residual lactase expression [2], ingestion with other dietary components [26], gut-transit time, small bowel bacterial overgrowth [22,23], and also composition of the enteric microbiome (e.g., high *vs.* low fermenters, hydrogen *vs.* methane producers) [25,27,28,29]. In addition to these environmental and physiological factors, it has been shown that patients with irritable bowel syndrome are at particular risk of both self-reporting dairy intolerance [9,30] and experiencing symptoms after lactose and FODMAP ingestion [31,32].

**Figure 3:**
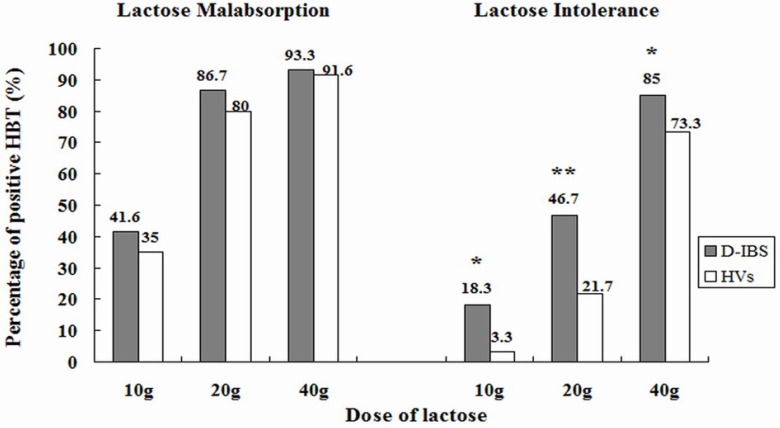
Prevalence of lactose malabsorption (LM) and lactose intolerance (LI) in patients with diarrhea predominant irritable bowel syndrome (IBS-D) and controls at 10-, 20-, and 40-g lactose hydrogen breath test (HBTs). * *p* < 0.05; ** *p* < 0.01 [31].

**Figure 4:**
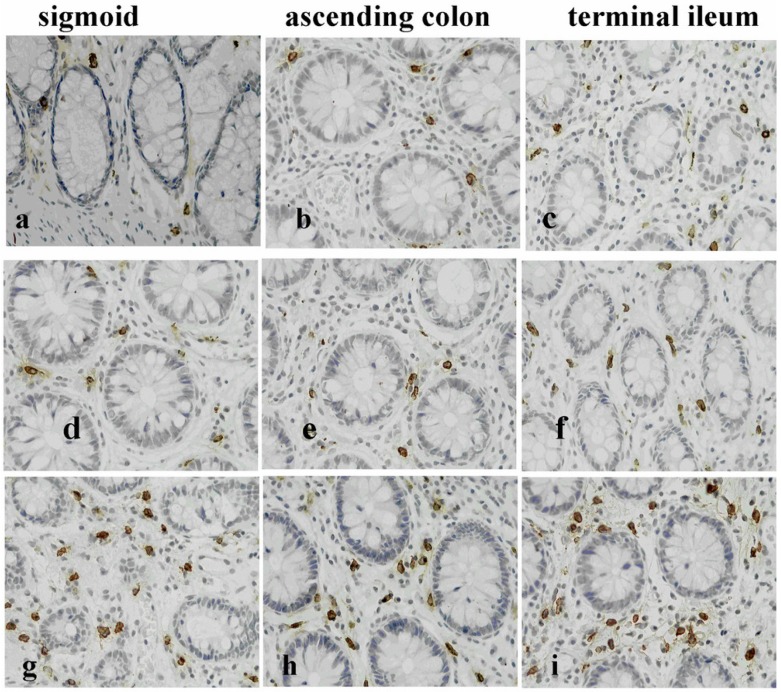
Representative photomicrographs showing tryptase positive mast cells (MCs) in the colonic mucosa of a healthy control (HCs) (**a**–**c**); an diarrhea predominant irritable bowel syndrome (IBS-D) patient with lactose malabsorption (LM) (**d**–**f**) and a patient with lactose intolerance (LI) (**g**–**i**). IBS-D patients with LI had increased mucosal MCs compared with LM and HCs [36].

Symptoms of lactose intolerance generally do not occur until there is less than 50% of lactase activity. Regular lactose intake may also have an effect. Although lactase expression is not up-regulated by lactose ingestion, tolerance could be induced by adaptation of the intestinal flora [26]. Further, most people with lactase non-persistence can tolerate small amounts of lactose (less than 12 g, equivalent to one cup), especially when it is combined with other foods or spread throughout the day [26,33]. A double-blinded, randomized, three-way cross over comparison of lactose tolerance testing at 10 g, 20 g and 40 g lactose was performed in patients with diarrhea predominant irritable bowel syndrome (IBS-D) and controls in a Chinese population with lactase deficiency [31]. The study design included a dose below normal symptom threshold (10 g), plus a dose reflecting normal intake at a single meal (20 g) and a “positive control” such as that used in epidemiological trials (40 g). The multiple-dose method (Figure 3) not only demonstrates the effect of dose in both study groups, but also guides nutritional management in a given patient. Importantly, the risk of symptoms in this study was greatly increased in IBS-D patients, especially at low-moderate doses found in the diet [31]. Indeed, few healthy controls with lactase non-persistence reported gastrointestinal (GI) symptoms except at the 40 g lactose dose [31]. IBS patients are known to be more sensitive to a variety of dietary and physical interventions that distend the GI tract [34]. Further studies in the same Chinese population demonstrated that anxiety, visceral hypersensitivity (defined by rectal barostat) and high-levels of gas production on breath tests are associated with patient reports of symptoms after ingestion of a modest (20 g) dose of lactose [35]. Heightened sensitivity to distension was associated with abdominal pain, bloating and overall symptom severity. Excessive gas production contributed to digestive symptoms, especially bloating and borborygmi [35]. Very interestingly, the same group of IBS patients that had lactose intolerance on hydrogen breath testing also had heightened activity of the innate mucosal immune system with increased counts of mast cells, intraepithelial lymphocytes and enterochrommafin cells in the terminal ileum and right colon (Figure 4), with release of pro-inflammatory cytokines after lactose ingestion [36]. These observations are similar to those seen in patients with post-infective IBS and provide insight into the pathophysiological basis of food intolerance [37].

Another condition that may play a role in food tolerance is small intestinal bacterial overgrowth (SIBO) caused by abnormally high bacterial counts in the small intestine, exceeding 10^5^ organisms/mL [38]. SIBO is clinically characterized by bloating, abdominal discomfort and diarrhea, symptoms that are very comparable to those of lactose intolerance [39]. Bacterial fermentation of lactose with production of short-chain fatty acids and gas in the small bowel may be particularly likely to trigger abdominal symptoms. Consistent with this hypothesis, combined scintigraphy and breath test studies showed a higher prevalence of SIBO in IBS patients with lactose intolerance than in the lactose malabsorption control group [22]. This effect appeared to be independent of oro-caecal transit time and visceral sensitivity [22].

## 4. Clinical Diagnosis of Lactose Malabsorption and Intolerance

**Figure 5:**
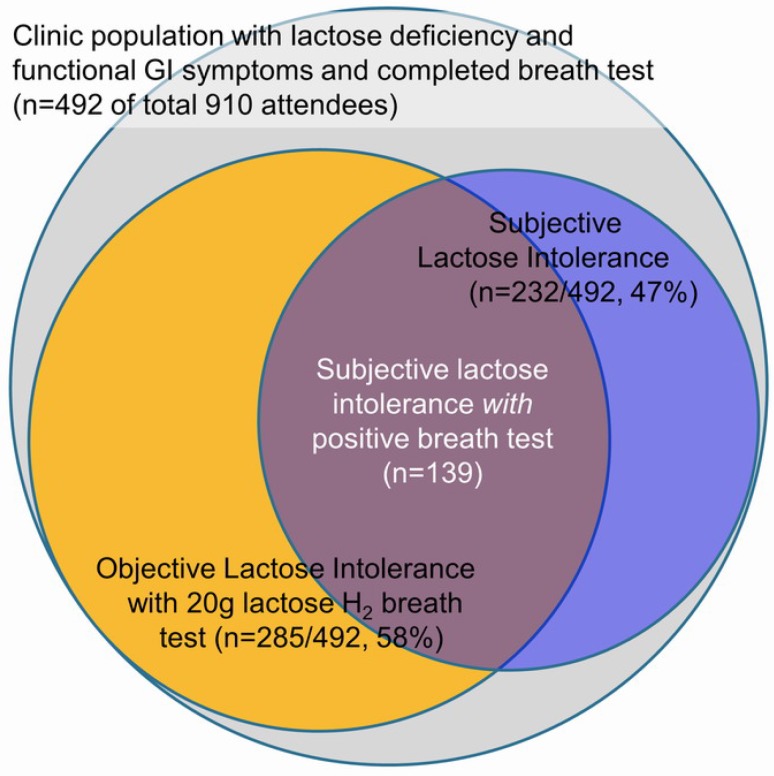
Lack of agreement between objective and subjective assessment of lactose intolerance [9].

Problems with lactose absorption have been described, detected and diagnosed in several ways and this can lead to confusion among doctors and patients [26]. Lactase deficiency is defined as markedly reduced brush-border lactase activity relative to the activity observed in infants. Lactose malabsorption occurs when a substantial amount of lactose is not absorbed in the intestine. Because lactose malabsorption is nearly always attributable to lactase deficiency, the presence of this condition can be inferred from measurements of lactose malabsorption such as an increase of glucose in the blood or an increase of hydrogen in the breath. The term lactose intolerance is defined by patient reports of abdominal pain, bloating, borborygmi, and diarrhea induced by lactose. Less often it can present with nausea or constipation and a range of systemic symptoms, including headaches, fatigue, loss of concentration, muscle and joint pain, mouth ulcers, and urinary difficulties [40,41]; however, it is unclear whether these atypical symptoms are directly due to lactose ingestion, or related to the presence of so-called “functional diseases”, such as irritable bowel syndrome (IBS), which is often accompanied by multiple somatic complaints. Certainly, it is not possible to make a definitive diagnosis on clinical presentation alone because double-blind trials have shown that the association of self-reported lactose intolerance and the occurrence of symptoms after lactose ingestion are very poor [42], even in patients with lactase deficiency (Figure 5) [9].

There are various methods (Table 1) for diagnosing lactose malabsorption and intolerance [25]. Testing of lactase activity in mucosal biopsies from the duodenum is regarded as the reference standard for primary and secondary lactase deficiency [43], however, limitations include the inhomogeneous expression of lactase [44] and the invasiveness of the test. Genetic tests may be useful for identifying lactase persistence in some European populations as the T-13910 allele is ~86%–98% associated with lactase persistence in European populations [5,6,7], however other SNPs are present in Arabian and African populations [10,11,12]. Future genetic tests will likely cover a range of genetic polymorphisms, potentially eliminating this limitation. A further limitation of both biopsy and genetic tests is that no assessment of symptoms is made. This impacts on the clinical relevance of these investigations because, as addressed above, only a proportion of patients with lactase deficiency develop abdominal symptoms after ingesting lactose [31].

**Table 1:** Summary of tests for lactose malabsorption and lactose tolerance [25].

	H_2_-Breath Test [17,45]	Lactose Tolerance Test [46]	Genetic Test [3,12]	Lactase Activity at Jejunal Brush Border [43,44]
**Test principle**	Increase of H_2_ in respiratory air after lactose challenge	Increase of blood sugar after lactose challenge	Genetic-13910C/T polymorphism	Enzymatic activity of lactase enzyme in biopsy sample
**Cut off**	>20 ppm within 3 h	<1.1 mmol/L within 3 h	C:C13910 lactase non-persistence	<17–20 IU/g
**Availability**	Good	Excellent	Variable	Rare
**False positives** (incorrect diagnosis)	Rapid GI-transit, small-intestinal bacterial overgrowth	Rapid GI-transit, impaired glucose tolerance	Rare (<5%) in Caucasians	Probably rare
**False negatives** malabsorption wrongly excluded	Non-H_2_-producers. Full colonic adaptation	Fluctuations in blood sugar	All causes of secondary lactose malabsorption	Patchy enzyme expression
**Secondary causes**	Cannot be excluded, kinetic of H_2_-increase can be suggestive	Cannot be excluded	Cannot be excluded	Can be excluded (histopathology at same procedure)
**Symptom assessment**	Possible	Possible	Not possible	Not possible
**Comment**	Method of choice for assessment of lactose malabsorption and intolerance	Rarely performed due to inferior sensitivity and specificity	Definitive in Caucasians. Less in other populations. Not suitable in secondary lactase deficiency.	Reference standard for detection of lactase deficiency (primary or secondary)
**Cost**	Low	Lowest	High	Highest

Lactose digestion and the association of maldigestion with symptoms can be assessed by the H_2_-breath test [45] and the lactose tolerance test [46]; however, the former is confounded by fluctuations of postprandial blood sugar. The H_2_-breath test can be false positive in the presence of small intestinal bacterial overgrowth; however, a larger problem is false-negative tests due to the presence of hydrogen non-producing bacteria in the colon (2%–43%) [17]. This problem of “hydrogen non-production” can be mitigated to some extent by examining patient reports of symptoms after the test dose. Patients with “false positive” breath tests complain of symptoms directly after ingestion. Those with “true positive” lactose intolerance complain of symptoms only after the substrate has entered the colon (usually 50–100 min). Another possibility is to combine the biopsy or genetic test (in Caucasians) with the H_2_-breath test; however, this is an expensive and time-consuming approach.

## 5. Treatment of Lactose Intolerance

Treatment of lactose intolerance should not be primarily aimed at reducing malabsorption but rather at improving gastrointestinal symptoms. Restriction of lactose intake is recommended because in blinded studies patients with self-reported lactose intolerance, even those with IBS, can ingest at least 12 g lactose without experiencing symptoms [26,47]. Even larger doses (15 to 18 g lactose) appear to be tolerated when dairy products are taken with other nutrients [26]. One retrospective case review reported improvement of abdominal discomfort, with lactose restriction in up to 85% of IBS patients with lactose malabsorption [48]; however, prospective studies show that lactose restriction alone is not sufficient for effective symptom relief in functional GI disease [49]. In our experience this approach is effective if symptoms are related only to dairy products; however, in IBS patients, lactose intolerance tends to be part of a wider intolerance to poorly absorbed, fermentable oligo-, di-, monosaccharides and polyols (FODMAPs) [9,30]. Evidence from recent trials indicates that this is present in about half of patients with IBS and this group requires not only restriction of lactose intake, but also a low FODMAP diet to improve gastrointestinal complaints. An initial controlled trial of a diet low in FODMAPs reported symptom improvement in 86% of IBS patients, compared to 49% for a standard dietary intervention [50]. Three randomized controlled trials have confirmed that a low FODMAP diet can benefit a wide range of symptoms in IBS patients [32,51,52]. All these studies included lactose restriction in the early “strict” phase of the dietary intervention; however, the specific role of lactose in causing symptoms was not assessed. A major issue with almost all dietary intervention trials is that the contribution of individual components (e.g. lactose) is difficult to assess as other dietary components (e.g., fat [53]) can also produce symptoms and, potentially, confound results.

Lactase enzyme replacement is another important approach in patients with “isolated” lactose intolerance that wish to enjoy dairy products. One double-blind, placebo-controlled, crossover study shows that in lactose malabsorbers with intolerance, lactase obtained from *Kluyveromyces lactis* represents a valid therapeutic strategy, with objective and subjective efficacy and without side effects [54]. Exogenous lactase obtained from *Aspergillus oryzae* or from *Kluyveromyces lactis* breaks down lactose into glucose and galactose to allow an efficient absorption [55].

A related strategy involves probiotics that alter the intestinal flora and may have beneficial effects in IBS patients [56]. Four-week consumption of a probiotic combination of *Lactobacillus casei Shirota* and *Bifidobacterium breve Yakult* improved symptoms and decreased hydrogen production in lactose intolerant patients. These effects appeared to persist for at least three months after suspension of probiotic consumption [56]. However, in another study, milk containing *Lactobacillus acidophilus* did not consistently reduce gastrointestinal symptoms in patients with self-reported lactose intolerance compared with control participants [26]. Further studies are required to provide high quality evidence to support or compare the efficacy of these strategies.

## 6. Long-Term Effects of Lactose or FODMAP Restriction

Although restricting dietary lactose or FODMAPs may improve gastrointestinal complaints, long-term effects of a diet free of dairy or FODMAPs products may be of concern [57]. Dairy products are the major source of calcium in many individuals. No study has addressed the safety and effectiveness of calcium replacement for patients with lactose intolerance; however, it seems reasonable to recommend increasing calcium intake from other foods or supplements in patients that restrict intake of dairy products, especially in the presence of other risk factors for osteoporosis.

Diet also has effects on the colonic microbiome. Altering the dietary intake of FODMAPs alter gastrointestinal microbiota [58] and a significant decrease in the concentration of probiotic *bifidobacteria* after four weeks of a low FODMAP diet has been reported [52]. Whether this change has any long-term implications is unknown. Recommending alternative foods is a key component of patient education and even with dietetic advice nutrient intake, in particular of calcium, can be compromised on a low lactose, low FODMAP diet.

Another issue that should be considered is the negative effect of dietary restriction on quality of life [9,59]. Patients with self-reported lactose intolerance restrict intake not only of dairy products but also of other foodstuffs due to general concerns about diet and health [9,59]. This is stressful and can be expensive as shown by the recent trend to “gluten free diets” [60]. Moreover, if not properly supervised, multiple food restrictions could lead to mal- or under-nutrition. Formal dietary intervention excludes a wide range of potential dietary triggers for a short period to achieve symptom improvement, followed by gradual food reintroduction to identify items and threshold doses that can be tolerated by patients.

## 7. Conclusions

Primary lactase deficiency can be regarded as the commonest “genetic disease” in the World, although, in truth, loss of lactase expression in adulthood represents the normal “wild-type” and lactase persistence the abnormal “mutant” state. Additionally, in secondary lactase deficiency, the ability to digest lactose can be lost due to infection, surgery and other insults. Whatever the cause, lactose malabsorption causes symptoms by several mechanisms: unabsorbed lactose leads to osmotic diarrhea; products of its bacterial digestion lead to secretory diarrhea and gas can distend the colon. Diagnosis of lactose malabsorption is based on detection either of the genetic mutation, loss of lactase activity in the enteric mucosa or evidence of malabsorption in the blood or breath. However, the presence of lactose malabsorption does not necessarily imply that abdominal symptoms are related to this process. The majority of healthy individuals with lactase deficiency tolerate up to 20 g lactose without difficulty. Instead, diagnosis of lactose intolerance requires concurrent assessment of lactose digestion and abdominal symptoms.

Recent studies have provided important new insight into the complex relationship between lactase deficiency, lactose malabsorption and symptom generation. This work has shed light on the wider issue of food intolerance as a cause of symptoms in irritable bowel syndrome and related conditions. Understanding the biological mechanism for food intolerance to lactose and FODMAPs will help clinicians make a definitive diagnosis and guide rational dietary and medical management. Ongoing studies will provide high quality evidence to document the efficacy and long-term effects of these strategies.

## References

[1] Vesa T.H., Marteau P., Korpela R. (2000). Lactose intolerance. J. Am. Coll. Nutr..

[2] Swallow D.M. (2003). Genetics of lactase persistence and lactose intolerance. Ann. Rev. Genet..

[3] Enattah N.S., Sahi T., Savilahti E., Terwilliger J.D., Peltonen L., Jarvela I. (2002). Identification of a variant associated with adult-type hypolactasia. Nat. Genet..

[4] Wang Y., Harvey C.B., Pratt W.S., Sams V.R., Sarner M., Rossi M., Auricchio S., Swallow D.M. (1995). The lactase persistence/non-persistence polymorphism is controlled by a *cis*-acting element. Hum. Mol. Genet..

[5] Poulter M., Hollox E., Harvey C.B., Mulcare C., Peuhkuri K., Kajander K., Sarner M., Korpela R., Swallow D.M. (2003). The causal element for the lactase persistence/non-persistence polymorphism is located in a 1 Mb region of linkage disequilibrium in Europeans. Ann. Hum. Genet..

[6] Hogenauer C., Hammer H.F., Mellitzer K., Renner W., Krejs G.J., Toplak H. (2005). Evaluation of a new DNA test compared with the lactose hydrogen breath test for the diagnosis of lactase non-persistence. Eur. J. Gastroenterol. Hepatol..

[7] Ridefelt P., Hakansson L.D. (2005). Lactose intolerance: Lactose tolerance test *versus* genotyping. Scand. J. Gastroenterol..

[8] Sun H.M., Qiao Y.D., Chen F., Xu L.D., Bai J., Fu S.B. (2007). The lactase gene-13910T allele can not predict the lactase-persistence phenotype in north China. Asia Pac. J. Clin. Nutr..

[9] Zheng X., Chu H., Cong Y., Deng Y., Long Y., Zhu Y., Pohl D., Fried M., Dai N., Fox M. (2015). Self-reported lactose intolerance in clinic patients with functional gastrointestinal symptoms: Prevalence, risk factors, and impact on food choices. Neurogastroenterol. Motil..

[10] Imtiaz F., Savilahti E., Sarnesto A., Trabzuni D., Al-Kahtani K., Kagevi I., Rashed M.S., Meyer B.F., Jarvela I. (2007). The T/G 13915 variant upstream of the lactase gene (LCT) is the founder allele of lactase persistence in an urban Saudi population. J. Med. Genet..

[11] Ingram C.J., Elamin M.F., Mulcare C.A., Weale M.E., Tarekegn A., Raga T.O., Bekele E., Elamin F.M., Thomas M.G., Bradman N. (2007). A novel polymorphism associated with lactose tolerance in Africa: Multiple causes for lactase persistence?. Hum. Genet..

[12] Tishkoff S.A., Reed F.A., Ranciaro A., Voight B.F., Babbitt C.C., Silverman J.S., Powell K., Mortensen H.M., Hirbo J.B., Osman M. (2007). Convergent adaptation of human lactase persistence in Africa and Europe. Nat. Genet..

[13] Wray G.A., Hahn M.W., Abouheif E., Balhoff J.P., Pizer M., Rockman M.V., Romano L.A. (2003). The evolution of transcriptional regulation in eukaryotes. Mol. Biol. Evol..

[14] Enattah N.S., Kuokkanen M., Forsblom C., Natah S., Oksanen A., Jarvela I., Peltonen L., Savilahti E. (2007). Correlation of intestinal disaccharidase activities with the C/T-13910 variant and age. World J. Gastroenterol..

[15] Ingram C.J., Mulcare C.A., Itan Y., Thomas M.G., Swallow D.M. (2009). Lactose digestion and the evolutionary genetics of lactase persistence. Hum. Genet..

[16] Itan Y., Jones B.L., Ingram C.J., Swallow D.M., Thomas M.G. (2010). A worldwide correlation of lactase persistence phenotype and genotypes. BMC Evol. Biol..

[17] Gasbarrini A., Corazza G.R., Gasbarrini G., Montalto M., di Stefano M., Basilisco G., Parodi A., Usai-Satta P., Vernia P., Anania C. (2009). Methodology and indications of H2-breath testing in gastrointestinal diseases: The Rome Consensus Conference. Aliment. Pharmacol. Ther..

[18] Magge S., Lembo A. (2012). Low-FODMAP Diet for Treatment of Irritable Bowel Syndrome. Gastroenterol. Hepatol..

[19] Shepherd S.J., Lomer M.C., Gibson P.R. (2013). Short-chain carbohydrates and functional gastrointestinal disorders. Am. J. Gastroenterol..

[20] Madsen J.L., Linnet J., Rumessen J.J. (2006). Effect of nonabsorbed amounts of a fructose-sorbitol mixture on small intestinal transit in healthy volunteers. Dig. Dis. Sci..

[21] Murray K., Wilkinson-Smith V., Hoad C., Costigan C., Cox E., Lam C., Marciani L., Gowland P., Spiller R.C. (2014). Differential effects of FODMAPs (fermentable oligo-, di-, mono-saccharides and polyols) on small and large intestinal contents in healthy subjects shown by MRI. Am. J. Gastroenterol..

[22] Zhao J., Fox M., Cong Y., Chu H., Shang Y., Fried M., Dai N. (2010). Lactose intolerance in patients with chronic functional diarrhoea: The role of small intestinal bacterial overgrowth. Aliment. Pharmacol. Ther..

[23] Zhao J., Zheng X., Chu H., Zhao J., Cong Y., Fried M., Fox M., Dai N. (2014). A study of the methodological and clinical validity of the combined lactulose hydrogen breath test with scintigraphic oro-cecal transit test for diagnosing small intestinal bacterial overgrowth in IBS patients. Neurogastroenterol. Motil..

[24] Croagh C., Shepherd S.J., Berryman M., Muir J.G., Gibson P.R. (2007). Pilot study on the effect of reducing dietary FODMAP intake on bowel function in patients without a colon. Inflamm. Bowel Dis..

[25] Misselwitz B., Pohl D., Fruhauf H., Fried M., Vavricka S.R., Fox M. (2013). Lactose malabsorption and intolerance: Pathogenesis, diagnosis and treatment. United Eur. Gastroenterol. J..

[26] Shaukat A., Levitt M.D., Taylor B.C., MacDonald R., Shamliyan T.A., Kane R.L., Wilt T.J. (2010). Systematic review: Effective management strategies for lactose intolerance. Ann. Intern. Med..

[27] Casen C., Vebo H.C., Sekelja M., Hegge F.T., Karlsson M.K., Ciemniejewska E., Dzankovic S., Froyland C., Nestestog R., Engstrand L. (2015). Deviations in human gut microbiota: A novel diagnostic test for determining dysbiosis in patients with IBS or IBD. Aliment. Pharmacol. Ther..

[28] He T., Priebe M.G., Zhong Y., Huang C., Harmsen H.J., Raangs G.C., Antoine J.M., Welling G.W., Vonk R.J. (2008). Effects of yogurt and *bifidobacteria* supplementation on the colonic microbiota in lactose-intolerant subjects. J. Appl. Microbiol..

[29] Zhong Y., Priebe M.G., Vonk R.J., Huang C.Y., Antoine J.M., He T., Harmsen H.J., Welling G.W. (2004). The role of colonic microbiota in lactose intolerance. Dig. Dis. Sci..

[30] Bohn L., Storsrud S., Simren M. (2013). Nutrient intake in patients with irritable bowel syndrome compared with the general population. Neurogastroenterol. Motil..

[31] Yang J., Deng Y., Chu H., Cong Y., Zhao J., Pohl D., Misselwitz B., Fried M., Dai N., Fox M. (2013). Prevalence and presentation of lactose intolerance and effects on dairy product intake in healthy subjects and patients with irritable bowel syndrome. Clin. Gastroenterol. Hepatol..

[32] Halmos E.P., Power V.A., Shepherd S.J., Gibson P.R., Muir J.G. (2014). A diet low in FODMAPs reduces symptoms of irritable bowel syndrome. Gastroenterology.

[33] Lomer M.C., Parkes G.C., Sanderson J.D. (2008). Review article: Lactose intolerance in clinical practice—Myths and realities. Aliment. Pharmacol. Ther..

[34] Spiller R., Aziz Q., Creed F., Emmanuel A., Houghton L., Hungin P., Jones R., Kumar D., Rubin G., Trudgill N. (2007). Guidelines on the irritable bowel syndrome: Mechanisms and practical management. Gut.

[35] Zhu Y., Zheng X., Cong Y., Chu H., Fried M., Dai N., Fox M. (2013). Bloating and distention in irritable bowel syndrome: The role of gas production and visceral sensation after lactose ingestion in a population with lactase deficiency. Am. J. Gastroenterol..

[36] Yang J., Fox M., Cong Y., Chu H., Zheng X., Long Y., Fried M., Dai N. (2014). Lactose intolerance in irritable bowel syndrome patients with diarrhoea: The roles of anxiety, activation of the innate mucosal immune system and visceral sensitivity. Aliment. Pharmacol. Ther..

[37] Spiller R., Garsed K. (2009). Postinfectious irritable bowel syndrome. Gastroenterology.

[38] Donaldson R.M. (1964). Normal Bacterial Populations of the Intestine and Their Relation to Intestinal Function. N. Engl. J. Med..

[39] Singh V.V., Toskes P.P. (2004). Small Bowel Bacterial Overgrowth: Presentation, Diagnosis, and Treatment. Curr. Treat. Options Gastroenterol..

[40] Campbell A.K., Wann K.T., Matthews S.B. (2004). Lactose causes heart arrhythmia in the water flea Daphnia pulex. Comp. Biochem. Physiol. Part B Biochem. Mol. Biol..

[41] Matthews S.B., Campbell A.K. (2000). When sugar is not so sweet. Lancet.

[42] Suarez F.L., Savaiano D.A., Levitt M.D. (1995). A comparison of symptoms after the consumption of milk or lactose-hydrolyzed milk by people with self-reported severe lactose intolerance. N. Engl. J. Med..

[43] Newcomer A.D., McGill D.B., Thomas P.J., Hofmann A.F. (1975). Prospective comparison of indirect methods for detecting lactase deficiency. N. Engl. J. Med..

[44] Maiuri L., Raia V., Potter J., Swallow D., Ho M.W., Fiocca R., Finzi G., Cornaggia M., Capella C., Quaroni A. (1991). Mosaic pattern of lactase expression by villous enterocytes in human adult-type hypolactasia. Gastroenterology.

[45] Metz G., Jenkins D.J., Peters T.J., Newman A., Blendis L.M. (1975). Breath hydrogen as a diagnostic method for hypolactasia. Lancet.

[46] Arola H. (1994). Diagnosis of hypolactasia and lactose malabsorption. Scand. J. Gastroenterol. Suppl..

[47] Savaiano D.A., Boushey C.J., McCabe G.P. (2006). Lactose intolerance symptoms assessed by meta-analysis: A grain of truth that leads to exaggeration. J. Nutr..

[48] Bohmer C.J., Tuynman H.A. (2001). The effect of a lactose-restricted diet in patients with a positive lactose tolerance test, earlier diagnosed as irritable bowel syndrome: A 5-year follow-up study. Eur. J. Gastroenterol. Hepatol..

[49] Parker T.J., Woolner J.T., Prevost A.T., Tuffnell Q., Shorthouse M., Hunter J.O. (2001). Irritable bowel syndrome: Is the search for lactose intolerance justified?. Eur. J Gastroenterol. Hepatol..

[50] Staudacher H.M., Whelan K., Irving P.M., Lomer M.C. (2011). Comparison of symptom response following advice for a diet low in fermentable carbohydrates (FODMAPs) *versus* standard dietary advice in patients with irritable bowel syndrome. J. Hum. Nutr. Diet..

[51] Ong D.K., Mitchell S.B., Barrett J.S., Shepherd S.J., Irving P.M., Biesiekierski J.R., Smith S., Gibson P.R., Muir J.G. (2010). Manipulation of dietary short chain carbohydrates alters the pattern of gas production and genesis of symptoms in irritable bowel syndrome. J. Gastroenterol. Hepatol..

[52] Staudacher H.M., Lomer M.C., Anderson J.L., Barrett J.S., Muir J.G., Irving P.M., Whelan K. (2012). Fermentable carbohydrate restriction reduces luminal bifidobacteria and gastrointestinal symptoms in patients with irritable bowel syndrome. J. Nutr..

[53] Simren M., Abrahamsson H., Bjornsson E.S. (2007). Lipid-induced colonic hypersensitivity in the irritable bowel syndrome: The role of bowel habit, sex, and psychologic factors. Clin. Gastroenterol. Hepatol..

[54] Montalto M., Nucera G., Santoro L., Curigliano V., Vastola M., Covino M., Cuoco L., Manna R., Gasbarrini A., Gasbarrini G. (2005). Effect of exogenous beta-galactosidase in patients with lactose malabsorption and intolerance: A crossover double-blind placebo-controlled study. Eur. J. Clin. Nutr..

[55] Ojetti V., Gigante G., Gabrielli M., Ainora M.E., Mannocci A., Lauritano E.C., Gasbarrini G., Gasbarrini A. (2010). The effect of oral supplementation with Lactobacillus reuteri or tilactase in lactose intolerant patients: Randomized trial. Eur. Rev. Med. Pharmacol. Sci..

[56] Almeida C.C., Lorena S.L., Pavan C.R., Akasaka H.M., Mesquita M.A. (2012). Beneficial effects of long-term consumption of a probiotic combination of *Lactobacillus*
*casei Shirota* and *Bifidobacterium breve Yakult* may persist after suspension of therapy in lactose-intolerant patients. Nutr. Clin. Pract..

[57] Wilt T.J., Shaukat A., Shamliyan T., Taylor B.C., MacDonald R., Tacklind J., Rutks I., Schwarzenberg S.J., Kane R.L., Levitt M. (2010). Lactose intolerance and health. Evid. Rep. Technol. Assess..

[58] Halmos E.P., Christophersen C.T., Bird A.R., Shepherd S.J., Gibson P.R., Muir J.G. (2015). Diets that differ in their FODMAP content alter the colonic luminal microenvironment. Gut.

[59] Bohn L., Storsrud S., Tornblom H., Bengtsson U., Simren M. (2013). Self-reported food-related gastrointestinal symptoms in IBS are common and associated with more severe symptoms and reduced quality of life. Am. J. Gastroenterol..

[60] Farnetti S., Zocco M.A., Garcovich M., Gasbarrini A., Capristo E. (2014). Functional and metabolic disorders in celiac disease: New implications for nutritional treatment. J. Med. Food.

